# Postnatal Care of Newborns and Mothers in Developing Countries

**Published:** 2008-03

**Authors:** La Rue K. Seims

**Affiliations:** Program Research and Evaluation, Saving Newborn Lives Program, Save the Children-USA, 2000 L Street, NW, Ste. 500, Washington, DC 20036, USA

Sir,

I was glad to see the issue of the Journal of Health, Population and Nutrition, devoted to reproductive and newborn health. Programme research has shown that mothers can be influenced to seek care for themselves and their newborns by an integrated package of community-based interventions (1). Most newborns born at home, and their mothers, however, do not receive care in the first few days after birth when many newborn problems occur.

The Saving Newborn Lives Program (SNL) of Save the Children-USA, funded by the Bill and Melinda Gates Foundation, introduced a basic newborn and maternal services package in six countries from 2002 to December 2004, reaching 7,092,818 women aged 15–49 years and 1,177,089 livebirths. Projects were conducted either directly by Save the Children or through partner not-for-profit organizations for 18 months or less.

The primary areas supported by the SNL Program in expanding the use of proven newborn interventions were training 12,950 health workers in essential newborn care, behaviour change communication approaches to promote healthful practices for mothers and newborns, and social mobilization for tetanus immunization. The SNL Program promoted the message that both newborns and their mothers should receive care together.

SNL staff and local partners conducted baseline and end-of-project surveys using the 30-cluster sampling method in five countries and a combination of 30-cluster and Lot Quality Assurance Sampling (LQAS) methods in one country (Bolivia). The subsample of mothers delivering at home, on which the two tables (Table 1 and 2) are based, was as follows: Respondents were mothers with a child aged less than one year, except for Pakistan where mothers with a child aged less than two years were surveyed.

**Table 1:** Subsample of mothers delivering at home

Country	Baseline (n=8,536)	Final (n=5,716)
Bolivia	852	474
Malawi	263	250
Mali	186	140
Bangladesh	3,104	2,787
Nepal	716	616
Pakistan	3,415	1,449

**Table 2:** Mothers delivering at home and their newborns who received care within three days in six countries, baseline and end-of-project, 2002–2004

Country	Indicators					
	Proportion of mothers who delivered at home who received postpartum care within 3 days			Proportion of mothers whose infant was born at home and received newborn care within 3 days		
	Baseline %	End-of-project %	p value	Baseline %	End-of-project %	p value
Malawi	7 (3–11)	3 (0–6)	>0.05	3 (1–6)	4 (1–7)	>0.05
Mali	1 (0–3)	8 (2–14)	>0.05	4 (0–8)	26 (16–36)	<0.05
Bolivia	5 (3–7)	16 (11–21)	<0.05	14 (11–17)	30 (24–36)	<0.05
Bangladesh	5 (4–6)	23 (21–25)	<0.05	2 (1–3)	32 (30–34)	<0.05
Nepal	4 (2–6)	16 (12–20)	<0.05	3 (1–5)	17 (13–21)	<0.05
Pakistan	3 (2–4)	20 (17–23)	<0.05	7 (6–8)	22 (19–25)	<0.05

Confidence intervals shown

A standard error (SE) was computed around each baseline and end-of-project coverage proportion using the formula: SE=sqrt(deff*1.962*p*(1-p)/n).

This assumes a 95% confidence interval with a cluster design factor of 2. Changes from baseline to final were considered to be significant when the SEs around each measure did not overlap.

At baseline, a very few newborns born at home in any of the six countries (range: 2% in Bangladesh to 14% in Bolivia) received care within three days. The baseline coverage for mothers receiving postpartum care was similarly low. Care was defined as either a clinic-visit or a home-visit by a trained or skilled healthcare worker. The content of the care varied from country to country and even in different implementation areas within the same country. There were significant increases in the proportion of newborns receiving care from baseline to end-of-project in five of the six countries. For example, the rate doubled (14% to 30%) in Bolivia and increased 16-fold (2% to 32%) in Bangladesh within 18 months or less of programme interventions. However, at least two-thirds of all newborns still received no care within the first three days of their birth in all countries at end-of-project.

Despite the clear and rapid progress, much more needs to be done to deliver postpartum interventions to mothers and their newborns, including innovative strategies, to increase demand for, access to, and use of newborn and postpartum care, especially among high-risk populations.

## References

[1] Manandhar DS, Osrin D, Shrestha BP, Mesko N, Morrison J, Tumbahangphe KM (2004). Effect of a participatory intervention with women's groups on birth outcomes in Nepal: cluster-randomised controlled trial. Lancet.

